# Preferential, enhanced breast cancer cell migration on biomimetic electrospun nanofiber ‘cell highways’

**DOI:** 10.1186/1471-2407-14-825

**Published:** 2014-11-10

**Authors:** Mark Tyler Nelson, Aaron Short, Sara L Cole, Amy C Gross, Jessica Winter, Tim D Eubank, John J Lannutti

**Affiliations:** Department of Biomedical Engineering, Ohio State University, Columbus, OH USA; Campus Microscopy and Imaging Facility, Ohio State University, Columbus, OH USA; Department of Internal Medicine, Ohio State University, Columbus, OH USA; Department of Chemical and Biomolecular Engineering, Ohio State University, Columbus, OH USA; Department of Materials Science and Engineering, Ohio State University, 143 Fontana Labs, 116 W 19th Ave, Columbus, OH 43210-1179 USA

**Keywords:** Topography, Cell motility, Chemotaxis, MDA-MB-231, MCF-10A, MCF-7

## Abstract

**Background:**

Aggressive metastatic breast cancer cells seemingly evade surgical resection and current therapies, leading to colonization in distant organs and tissues and poor patient prognosis. Therefore, high-throughput *in vitro* tools allowing rapid, accurate, and novel anti-metastatic drug screening are grossly overdue. Conversely, aligned nanofiber constitutes a prominent component of the late-stage breast tumor margin extracellular matrix. This parallel suggests that the use of a synthetic ECM in the form of a nanoscale model could provide a convenient means of testing the migration potentials of cancer cells to achieve a long-term goal of providing clinicians an *in vitro* platform technology to test the efficacy of novel experimental anti-metastatic compounds.

**Methods:**

Electrospinning produces highly aligned, cell-adhesive nanofiber matrices by applying a strong electric field to a polymer-containing solution. The resulting fibrous microstructure and morphology closely resembles *in vivo* tumor microenvironments suggesting their use in analysis of migratory potentials of metastatic cancer cells. Additionally, a novel interface with a gel-based delivery system creates CXCL12 chemotactic gradients to enhance CXCR4-expressing cell migration.

**Results:**

Cellular dispersions of MCF-10A normal mammary epithelial cells or human breast cancer cells (MCF-7 and MDA-MB-231) seeded on randomly-oriented nanofiber exhibited no significant differences in total or net distance traveled as a result of the underlying topography. Cells traveled ~2-5 fold greater distances on aligned fiber. Highly-sensitive MDA-MB-231 cells displayed an 82% increase in net distance traversed in the presence of a CXCL12 gradient. In contrast, MCF-7 cells exhibited only 31% increase and MCF-10A cells showed no statistical difference versus control or vehicle conditions. MCF-10A cells displayed little sensitivity to CXCL12 gradients, while MCF-7 cells displayed early sensitivity when CXCL12 concentrations were higher. MDA-MB-231 cells displayed low relative expression levels of CXCR4, but high sensitivity resulting in 55-fold increase at late time points due to CXCL12 gradient dissipation.

**Conclusions:**

This model could create clinical impact as an *in vitro* diagnostic tool for rapid assessment of tumor needle biopsies to confirm metastatic tumors, their invasiveness, and allow high-throughput drug screening providing rapid development of personalized therapies.

**Electronic supplementary material:**

The online version of this article (doi:10.1186/1471-2407-14-825) contains supplementary material, which is available to authorized users.

## Background

One in eight women in the United States will develop malignant breast cancer in her lifetime [1]. Current therapies for triple negative breast cancer (TNBC) remain surgery, chemotherapy, and/or radiotherapy [2–5]. However, highly-metastatic tumor cells can invade local tissues and intravasate blood vessels to establish distant metastases in spite of primary resection or treatment, resulting in a 20% mortality rate after 5 years [1, 6, 7]. Novel, experimental anti-metastatic drugs and compounds that specifically target metastatic cells are grossly overdue. Effective, personalized anti-metastatic diagnostic and therapeutic approaches have been impeded due to a lack of *in vitro* models that adequately recapitulate cell invasion/migration mechanisms [4, 8–10] to allow for rapid development of anti-metastatic drugs [11–16].

Each local and distant metastasis are multi-step processes that require cancer cells to leave the primary tumor by migrating through the dense extracellular matrix (ECM) within the tumor, at the tumor-stroma interface, and within the stroma allowing intravasation and downstream colonization [6, 17, 18]. In breast cancer, the microenvironment changes significantly from onset to late stage cancer [9, 19–22]. One of the most influential parameters that drives tumor cell migration and subsequent invasion of surrounding ECM is topography [23–26]. Aggressive cancer cells follow “the path of least resistance” to invade ECM and encounter distal blood or lymphatic vessels for intravasation [27–29]. Tumor-associated collagen signatures, specifically TACS-3, as described by Conklin et al., is characterized by radially-organized, highly-aligned collagen fibers/bundles located at the tumor-stroma interface potentially providing a topography that enables rapid stromal invasion [25, 30]. Clinical observation of these aligned collagen bundles oriented perpendicular to the tumor boundary using histological evaluation of patient biopsies correlates to poor prognosis and reduced treatment efficacy [25, 31].

*In vitro* models designed to assess tumor cell metastatic potential vary considerably and typically possess both advantages and disadvantages. Commonly used models (scratch or Boyden chamber assays) lack quantitative assessment of true cell migration or invasion, allow only limited imaging opportunities or are characterized by the absence of microstructural features commonly found in the tumor microenvironment [32–35]. More specifically, scratch and Boyden chamber assays are both intimately connected to cellular proliferation. The metric for analyzing migration in each assay requires quantification of the area of cell occupancy or the total number of cells present. Furthermore, in either system cells are adhered to flat, 2D substrates meaning that migration takes place on surfaces lacking topographical cues. *In vitro* assays that more closely recapitulate *in vivo* microenvironments can better assess qualitative and quantitative biological phenomenon of cancer cell migration and invasion, chemotherapeutic effectiveness, novel anti-metastatic drug development, and attempt to provide patients with personalized treatment options. Johnson et al. previously demonstrated that aligned electrospun nanofibers closely resemble the morphology and microstructure of white matter tracts in the brain known to facilitate rapid dispersion of glioma brain cancer cells [33, 36]. Electrospun nanofibers can exhibit fiber diameters having the same magnitude as hyaluronan fibers and provide sufficient adhesion allowing U251 glioma cells to migrate efficiently on these aligned “cell highways” [33] while little migration was observed on random nanofiber [33, 36, 37]. In addition to morphology of electrospun nanofiber, the pseudo-3D environment provides a fibrous microstructure similar to that of *in vivo* tissues [38–41].

In this report, aligned and randomly-oriented nanofiber arrays are integrated with a gel-based chemotaxis system to assess the migratory potential of MCF-10A mammary epithelial cells as well as MCF-7 and MDA-MB-231 breast cancer cell lines. As hypothesized, the more highly metastatic MDA-MB-231 cells [42] migrated further and at greater velocities relative to the less metastatic MCF-7 cells on aligned nanofiber as a result of topographic and chemotactic guidance. In contrast, migration on randomly-oriented nanofiber resulted in little difference in total or net distance traveled between cell lines. As a more direct corollary to clinical samples, xenograft MDA-MB-231 tumors from SCID mice were explanted and cultured on aligned nanofiber arrays, where quantitative assessment of tumor cell migration displayed significant increases in total and net migration in the presence of a CXCL12 gradient. Aligned nanofiber topography and CXCL12 chemotactic gradients significantly enhanced cellular migration. Such nanofiber assays could provide useful in evaluating the metastatic potential of tumor biopsies.

## Methods

### Electrospinning

Polycaprolactone (PCL, 80,000 MW, Sigma-Aldrich) nanofiber scaffolds were manufactured as before via electrospinning [36]. Briefly, a 5 w/w% solution of PCL in 1,1,1,3,3,3-hexafluoro-2-propanol (HFIP, >99% purity, Oakwood Chemicals Inc.) was prepared by continuous stirring at room temperature. This solution was placed in a 60 ml syringe with a 20 gauge blunt tip needle and electrospun using a high voltage DC power supply (Glassman High Voltage, Inc.) set to +16 kV, a 20 cm tip-to-substrate distance [43] and a 5 ml/hour flow rate. Nanofiber was deposited onto an aluminum collector rotating at 15 m/s, covered with tissue culture treated polystyrene (TCPS) until a 100 μm thick layer was achieved. PCL nanofiber coated TCPS was then placed in a vacuum overnight to insure removal of residual solvent [44], and then plasma treated (Harrick Plasma) to promote cellular attachment [36], and subsequently affixed to 24-well cell culture plates for cellular experiments. Finally, using gamma-irradiation (Sterigenics) the plates were sterilized. Prior to inoculating cells onto PCL nanofiber plates, 1 mL of PBS (Sigma-Aldrich) was added to each of the wells and aspirated after 30 min.

Electrospun PCL nanofiber microstructure and morphology was analyzed using a Sirion FEG scanning electron microscope (SEM) at an accelerating voltage of 5 kV. Aligned and randomly deposited PCL nanofiber scaffolds were affixed to aluminum mounts with double-sided carbon tape (SPI Supplies Inc.) and then gold-sputter coated. Images of nanofiber general orientation, morphology, and microstructure were taken and ImageJ used to determine degree of alignment and fiber diameter from 5 different randomly taken images.

### Gel-based chemotaxis

100 ng recombinant human (rh)CXCL12 (CXCL12/SDF-1α, R&D Systems Inc.) was mixed into 200 μL agarose and pipetted onto the wall of a 24-well plate culture well while the plate was tilted at a 45° angle to prevent the agarose from spreading across the bottom of the well before fully solidifying. The resulting gel body consists of a small ‘bead’ of approximately 3 mm in length at the edge of each well.

To test the protein release rate, BSA-conjugated fluorescein isothiocyanate (BSA-FITC, Sigma-Aldrich) was used as a model. Using the method previously stated above, 1 mg of BSA-FITC was mixed with agarose and added to 24-well plate culture wells. Release of the BSA-FITC was conducted by adding 1 mL of PBS to each well, and after each time point a 20 uL aliquot of the release solution (PBS containing released BSA-FITC) was removed and added to 180 uL of pure-PBS in a 96-well plate. After 24 hours, all aliquots taken and contained in the 96-well plate were examined using a fluorescent 96-well plate spectrometer (Spectra Max 190 Absorbance UV–VIS plate reader) at an excitation wavelength of 485 nm and the fluorescent intensity measured at the emission wavelength of 535 nm. Using a serial dilution in PBS, a calibration curve was then used to convert the data to mg/mL concentration and plotted relative to time (Additional file 1).

### Cellular methods and reagents

MCF-10A human normal mammary epithelial cells, MCF-7 (ATCC^®^ HTB-22™) and MDA-MB-231 (ATCC^®^ HTB-26™) human breast cancer cells were grown in an incubator under 5% CO_2_ using Dulbecco's Modified Eagle Medium: Nutrient Mixture F-12 (DMEM/F-12) media, supplemented with 10% fetal bovine serum (FBS) and 1% penicillin/streptomycin. In addition, explanted GFP-labeled MDA-MB-231 breast tumors from SCID mice (Jackson Laboratories, strain NOD.CB17-Prkdc < scid>/J, The Ohio State University Institutional Animal Care and Use Committee approved our study utilising breast tumours from SCID mice (protocol number 2011A00000077)) were used. All cells used in experiments were from the same passage (no greater than 5) and were stained just prior to migration using 0.4 μL of CellTracker™ Green CMFDA (5-chloromethylfluorescein diacetate) live cell dye (Invitrogen, Cat. # C2925, USA). Cellular motion was detected by exciting the dye using an exposure time of 50 msec at a laser excitation wavelength of 485 nm and detecting emission at 535 nm on an Olympus IX81 confocal time-lapse microscope. Cellular viability inoculated on tissue cultured polystyrene (TCPS) versus electrospun PCL nanofiber (aligned or randomly oriented) was assessed using an XTT cell viability assay (Roche, Cat. 11465015001) as per the manufacturer’s protocol.

### qRT-PCR for CXCR4 mRNA expression

The expression of CXCR4 mRNA (receptor for CXCL12) was analyzed over a 24 hour period for all three cell lines to determine if exposure to its CXCL12 ligand over time transcriptionally regulates CXCR4 gene expression. 1.5×10^5^ cells were inoculated in 24-well plates in triplicate with or without 100 ng of rhCXCL12 (R&D Systems, Inc; 350-NS-010). After 0, 2, 8, or 24 hours of culture, 1 mL of TRIzol reagent (Invitrogen) was added to lyse the cells and all lysate was collected and frozen in -20 C until further processing.

Purified RNA was obtained using the RNeasy Minikit (QIAGEN) as per the manufacturer’s protocol. The RNA pellets was allowed to dry for 15 min before 20 μL of nuclease-free water was added to each of the samples in preparation for spectroscopy examination (Nanodrop, Thermo Scientific). cDNA was synthesized using the Superscript First Strand Synthesis System (Invitrogen) and used for real-time polymerase chain reaction (PCR) using SYBR Green PCR Master Mix (Applied Biosciences). The following primers: hCXCR4 forward: 5’-AGCATGACGGACAAGTACAGG-3’ and hCXCR4 reverse: 5’-GATGAAGTCGGGAATAGTCAGC-3’ were designed using Primer Express, Version 3.0 software (ABI Prism; PerkinElmer Life and Analytical Sciences) and synthesized by Invitrogen. Data were analyzed according to the comparative threshold method and normalized against the GAPDH internal control transcript.

### Cell migration assay

#### Scratch/wound healing assay

MDA-MB-231 cells were plated to confluence in 6-well culture plates containing Dulbecco’s Modified Eagles Medium (DMEM), 10% FBS, and 1% penicillin/streptomycin/amphotericin B (PSA) for scratch assay observation. Using a pipette tip, three horizontal scratches were made across the diameter of the well. In addition, on the bottom/back of each well, a line perpendicular to the scratches was drawn with a permanent marker. The monolayer was washed one time with PBS to remove any floating cells generated by the scratching process and fresh media applied. Using an inverted microscope, photos at the intersection of each cell scratch and the line on the bottom of the plate were captured immediately (six images per well) and used as a reference point for the 16 h time point to determine percentage scratch coverage.

#### Boyden/transwell assay

A 24-well plate of 8.0 μm pore-size diameter polycarbonate (PC) membrane transwell inserts (Corning, Cat. # 3422, USA) were pretreated with complete DMEM/F12 culture medium prior to cell inoculation. Inserts were rinsed with 1% FBS DMEM/F12 cell culture medium 2× and 600 μL of depleted DMEM/F12 medium added to the lower compartment of each transwell insert. 50,000 cells suspended in 100 μL of DMEM/F12 medium (supplemented with 1% FBS) or 1.5 mg/mL col I matrix gel were added to the upper compartment of the transwell and allowed to adhere for 8 hours. After allowing the cells to adhere and become appropriately (protocol for “Transwell Permeable Supports,” Corning Inc.) serum starved, the transwells were moved to new wells containing DMEM/F12 medium (supplemented with 1% FBS) with or without 100 ng/mL CXCL12 chemokine. After 24 hours, transwells of each condition assessed in triplicate were removed, the upper compartment side of the PC membrane wiped clean using a cotton swab and washed with fresh DMEM/F12 culture medium and the backside or lower compartment side of the membrane imaged. Four images from each membrane were taken from representative locations, and the number of cells present was counted and the average number of cells per experimental condition was reported.

#### Electrospun nanofiber assay

In addition, 1.05×10^4^ cells per cm^2^ were inoculated on aligned or randomly oriented electrospun PCL nanofiber in a 24-well cell culture plate to evaluate the migratory potential of three different cell lines. 100 μL cell suspensions were added to each well in triplicate, with or without gel, and with or without gel loaded with rhCXCL12. Cells were allowed to adhere for 2 hour before 1 mL of culture medium was added to each of the wells, denoting the starting time at which time-lapse confocal microscopy began. Three imaging locations with in each of the wells were recorded and subsequently imaged every 30 min for 24 hours. After 24 hours, all of the images at each representative location were chronologically converted into a time-lapse video from which cell migratory analysis was conducted. Using the MTrackJ cell motion plugin for ImageJ, 25 randomly-selected cells from each video were tracked and the total distance of migration, net distance of migration, and velocity was recorded. The total distance of migration was determined as the sum of each individual cell displacement that occurred. Conversely, net distance of migration was calculated as the net sum of the individual cell vectors with respect to a reference or with respect to an initial position at time zero (when no gradient was present). Migration velocity was recorded as an average over the full 24 hour time period, and as the average velocity with respect to each time point throughout the 24-hour time-lapse period.

### Multi-photon imaging

100 micron sections of Met-1 murine breast tumor, created from cells orthotopically implanted into the number 4 mammary gland of wild type C57Bl/6 female mouse sample, were obtained using a vibratome. Image stacks were captured using an Olympus FV100MPE microscope equipped with a 25X XLPlan water immersion objective lens (1.05 N.A>) and a Mai Tai DeepSee Ti:Sapphire tunable laser (Spectra-Physics, Newport Corp.). Second Harmonic Generation (SHG) detection of collagen was performed at a wavelength of 950 nm while backscattered SHG signals were detected at 855 nm. An image series was captured using an optical zoom of 2×. Both image stacks are presented as a two dimensional image projections.

### Statistical analysis

All quantitative results are displayed as the average value ± the standard deviation. To determine statistical significance Minitab 16 statistical software was employed. Using a 95% confidence interval an ANOVA was conducted for each variable independently and as interacting factors and p-values less than 0.05 resulted in a null-hypothesis rejection. Further analysis was conducted using a Tukey’s comparison test to more thoroughly determine statistical significance between levels of different variables and their potential interactions using a 95% confidence interval.

## Results

### Microscopy analysis

**Figure 1:**
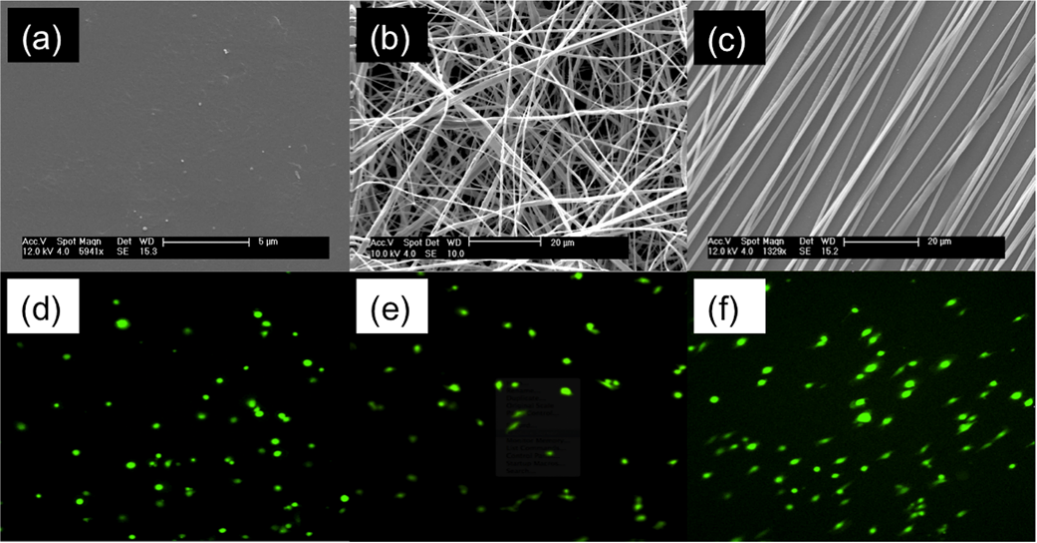
**Confocal microscopy images displaying breast cancer cell morphology. (a)** reveals the featureless morphology of standard, high modulus flat tissue culture polystyrene. **(b)** The morphology of random nanofibers as-spun. **(c)** The topography of relatively well-aligned nanofiber. Confocal images of MDA-MB-231 cells cultured on: **(d)** flat tissue culture polystyrene, random nanofiber **(e)** and **(f)** aligned nanofiber. The cells on polystyrene display no net alignment. On random nanofiber they display indecisive alignment, pointing in directions dictated by cell attachment to specific nanofibers. On aligned fiber the cells align in the direction of the underlying fiber and begin migrating.

**Figure 2:**
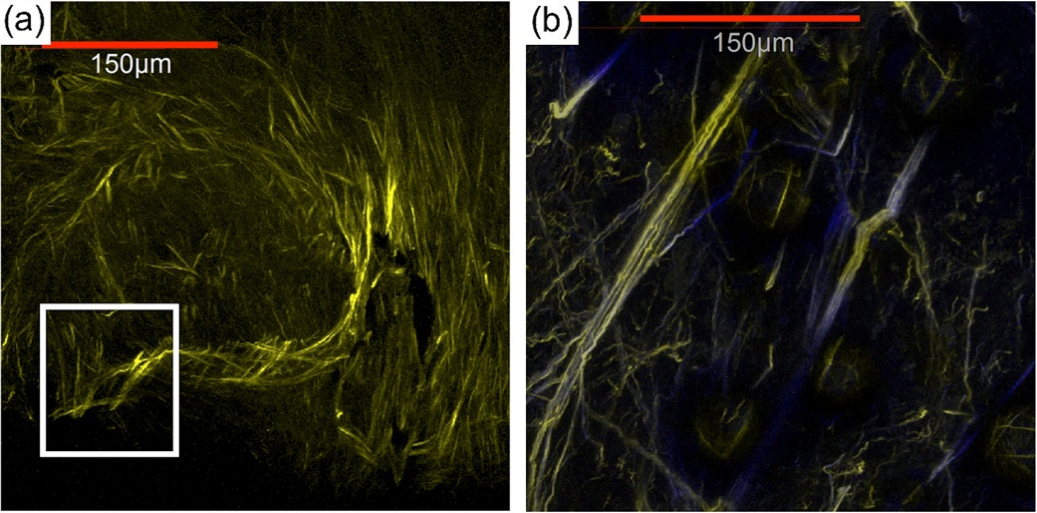
**Multiphoton microscopy images of collagen fibers from an explanted xenograft MDA-MB-231 tumor.** Images show dense collagen fibers near the central core **(a)** of a MDA-MB-231 xenograft breast tumor, and **(b)** radially aligned collagen fibers invading the tumor-stroma boundary.

Commonly used tissue culture polystyrene (TCPS) differs greatly in microstructure and morphology as compared to electrospun PCL nanofiber. Figure 1(a-c) depicts scanning electron microscopy (SEM) images of TCPS (a), randomly oriented (b), and aligned PCL nanofiber (c). TCPS displays a smooth, flat microstructure lacking micro-scale topographical features. In contrast, electrospun PCL nanofiber displays numerous nano- (fiber diameter) and micro-scale (inter-fiber pores) topographical features present within the tumor extracellular matrix (Figure 2). Figure 1(b) illustrates how randomly oriented fiber microstructure greatly differs from that of aligned nanofiber (a). Table 1 displays average alignment and fiber diameter for the random and aligned PCL nanofiber. No statistical difference was found between fiber diameters in Figure 1(b) random vs. Figure 1(c) aligned. On average, random fibers were 697 ± 58 nm in diameter. Aligned PCL nanofibers displayed slightly larger fiber diameters of 795 ± 125 nm. Significant differences (p < .005) were observed comparing fiber alignment in Figure 1(b) to that of Figure 1(c). 87.7% of the nanofibers seen in Figure 1(c) are aligned in the same direction, while only 5.6% were considered within 5 degrees of each other in the random fiber image (Figure 1(b)).

**Table 1:** **Quantitative information on fiber alignment (based on 25 measurements) and average diameter (based on 25 measurements)**

	Alignment	Fiber diameter (nm)
Aligned PCL	87.7 ± 5%***	795.4 ± 125
Random PCL	5.6 ± 45%	697.9 ± 58

***Denotes statistical significance, p = .0002.

Confocal images of MDA-MB-231 breast cancer cells were captured to illustrate differences in cell morphology observed on different substrates. Cells grown on TCPS (Figure 1(d)) display a rounded, spherical shape and exhibit both flat lamellipodia and filopodia. Figure 1(e) displays MDA-MB-231 cells grown onto randomly-oriented nanofiber where they exhibit a mixture of spherical and highly elongated cells. These cells appear to adhere to single PCL fibers (unhindered by neighboring fibers) to result in elongated, spindle-shaped cell morphologies. At the same time, cells which adhere and sit atop intersecting fibers display a more spherical morphology. In Figure 1(f) the high degree of alignment suggests that few such intersections exist, therefore the majority of these cells (>95%) display this elongated shape in the direction of fiber alignment.

Figure 2(a, b) demonstrates multi-photon, second harmonic generated (SHG) images of collagen I fibers located within explanted Met-1 mouse breast tumors resected from orthotopically-implanted tumor-bearing mice. Near the core of the tumor (Figure 2(a)), collagen fibers (yellow) are dense and display a variety of orientations. It appears that these fibers are more randomly oriented within the tumor stroma. At the tumor-stroma boundary, fibers are organized into aligned microstructures and radially oriented away from the center of the tumor. Arrows indicate a marked number of radially aligned collagen fibers oriented perpendicularly to the tumor-stroma boundary (Figure 2(b)). These collagen fibers are highly aligned and well organized to provide preferential, enhanced migration of metastatic tumor cells [25, 31].

### Cellular migration

**Figure 3:**
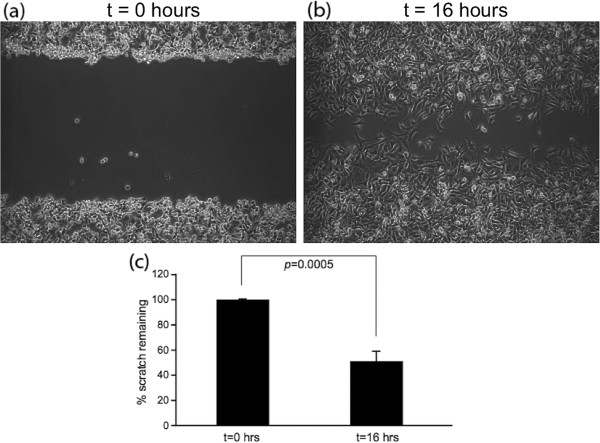
**Scratch assay data detailing the migration of cells out from a confluent monolayer onto a featureless ‘scratch’.** At time = 0 h **(a)** confluent MDA-MB-231 cells were scratched presenting at 100 μm wide gap with 100% area remaining. **(b,c)** After 16 hours only 48% of the original area remains.

**Figure 4:**
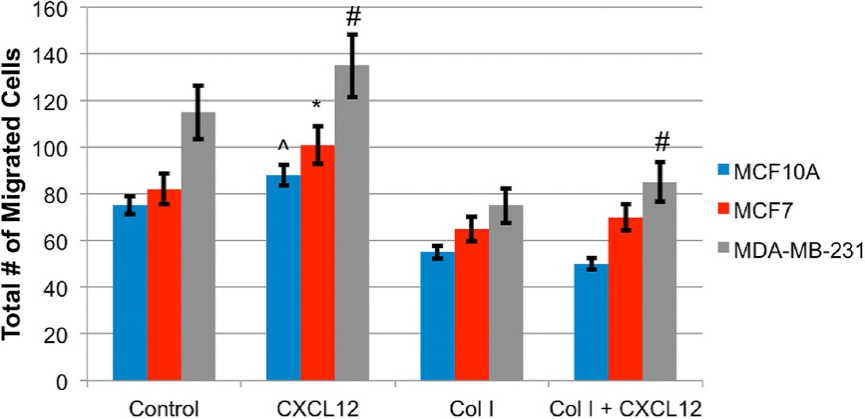
**Boyden chamber data showing the number of cells present on the lower compartment of a transwell membrane after 24 h.** MDA-MB-231 cells displayed the greatest number of cells present. On average MDA-MB-231 exhibited a 29% increase in cell count in the presence of the CXCL12 gradient. The collagen I gel barrier proved to discriminate against the MCF10A and MCF7 cells migratory ability more so than the MDA-MB-231 cells. Symbols denote statistically significant differences between experimental conditions, p <0.05. (#) for MDA-MB-231, (*) for MCF7, and (^) for MCF10A.

Figure 3(a-c) illustrates a traditional scratch/wound healing assay evaluating MDA-MB-231 cellular migration on TCPS. MDA-MB-231 cells were cultured to confluence in serum-containing media on TCPS at the start of the experiment (Figure 3(a)). After 16 hours, only 48% of the scratch area remains (Figure 3(b)). A total distance of 100 *μ* m spans the exposed area, limiting the net quantitative information obtainable.Boyden or transwell chambers are widely used to evaluate cell migration in response to a chemotactic gradient. MCF10A, MCF7 and MDA-MB-231 cells were inoculated on these porous, 2D substrates with and without the presence of a collagen matrix as an invasive barrier to assess their migration through 8 μm pores in response to the applied CXCL12 gradient. Figure 4 displays the cell counts for each cell line after 24 h. MCF10A cells displayed migrated cell counts statistically identical to that of MCF7 cells. However, MDA-MB-231 cells displayed 50 and 32% greater cell counts for the control and collagen I conditions, respectively. In the presence of CXCL12 the MCF10A cells displayed a 13% increase, the MCF7 cells a 28% increase, and MDA-MB-231 cells a 29% increase in the number of cells present on the backside of the membrane. When a collagen I barrier + CXCL12 gradient are present, the MCF10A and MCF7 cells display no statistical difference in counts while MDA-MB-231 cells display a 29% increase over migration through collagen in the absence of a CXCL12 gradient.

**Figure 5:**
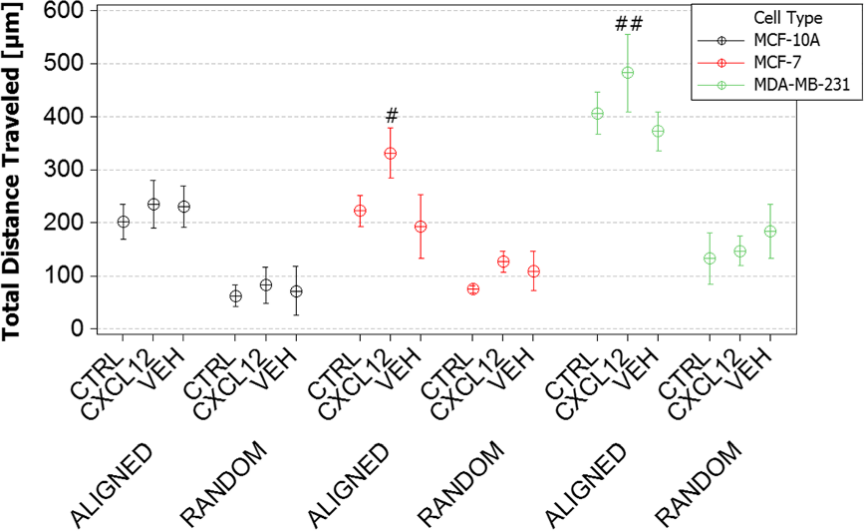
**Total distance traveled of MDA-MB-231, MCF-7 and MCF-10A cells on aligned and random fibers in the absence of a gel (‘control’), in the presence of a gel (‘vehicle’) and versus exposure to a gel-based CXCL-12 gradient (‘CXCL12’).** MCF-10A cells traveled approximately 136% farther on aligned versus random fiber but were not sensitive to the presence of the gradient. The total distance traveled by MCF-7 cells was very similarly governed by aligned versus random fiber topographies however the presence of the CXCL-12 gradient resulted in an average 1.5-fold increase. The total distance traveled by MDA-MB-231 cells on random fiber was very similar to that of the MCF-7 and MCF-10A cells (and was similarly unaffected by the presence of the gradient) but total travel on aligned fiber was at 87% greater that either MCF line. Total MDA-MB-231 travel in the presence of the chemical gradient was not statistically different than in the absence of the gradient. (time-lapse video footage of cell migration on random and aligned nanofiber substrates, Additional files 2 and 3). (#) Denotes statistically significant differences between experimental conditions, p <0.05.

For each cell line, Figure 5 displays total distance traveled versus random or aligned PCL nanofiber with or without the presence of the CXCL12 chemotactic gradient. In all instances, cells seeded on aligned nanofiber display significantly greater travel than the same cells inoculated on randomly oriented nanofiber (Additional files 2 and 3). In addition, no significant differences in the total distance traveled were observed on random fiber with or without the presence of CXCL12, for each of the cell lines. MCF-10A cells inoculated on aligned fiber displayed no significant difference in total migration when comparing either control vs. vehicle or in the presence of a CXCL12 gradient. However, they migrated 136% further compared to MCF-10A cells inoculated on random fiber. Likewise, MCF-7 cells displayed a 98% and MDA-MB-231 cells a 145% increase in total distance traveled on aligned fiber vs. randomly oriented PCL nanofiber. MCF-7 cells inoculated on aligned fiber in the presence of CXCL12 displayed a 61% increase in total distance traveled compared to either control or vehicle. MDA-MB-231 cells migrating on aligned nanofiber displayed a 36 and 113% increase in total distance traveled over MCF-7 and MCF-10A cell lines, respectively.

**Figure 6:**
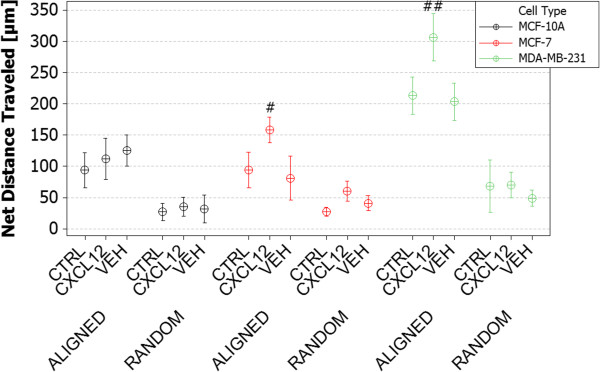
**Net distance traveled of MDA-MB-231, MCF-7 and MCF-10A cells on aligned and random fibers in the absence of a gel (‘control’), in the presence of a gel (‘vehicle’) and versus exposure to a gel-based CXCL-12 gradient (‘CXCL12’).** Observed trends are very similar to those of Figure 5 except that net MDA-MB-231 travel is 45% greater and statistically different from that of the control or the vehicle. (time-lapse video footage of cell migration on aligned nanofiber with applied CXCL12 chemotactic gradient, Additional file 4). (#) Denotes statistically significant differences between experimental conditions, p <0.05.

Figure 6 displays the results for net distance traveled for each of the cell lines, with respect to direction of a CXCL12 gradient (Additional file 4). As seen previously in Figure 5, no significant difference in net distance traveled was observed between the different cell lines when inoculated on randomly oriented PCL nanofiber regardless of the presence of a CXCL12 gradient. Significant (p < .05) 1.5-fold increases in net distance traveled were observed when comparing MCF-7 cells migrating in the presence of a CXCL12 gradient vs. MCF-10A or MCF-7 control or vehicle conditions. While MDA-MB-231 cells did not show any significant statistical difference in total distance traveled on aligned fiber in Figure 5, significant differences were observed when examining net distance traveled (Figure 6). MDA-MB-231 cells migrated 43% further on aligned fiber in the presence of a CXCL12 gradient compared to control or vehicle conditions, a 392% increase over cells seeded on randomly aligned nanofiber. In addition, MDA-MB-231 cells migrated 82% further in the presence of a CXCL12 gradient compared to MCF-7 or MCF-10A cells on aligned PCL nanofiber.

Table 2 displays the initial (average velocity between 0 to 8 h) and final (average velocity between 16 to 24 h) velocities for each of the cell lines (MCF-10A, MCF-7, and MDA-MB-231) inoculated on random or aligned nanofiber and with or without the presence of CXCL12 gradients. No significant differences in the V_i_ vs. V_f_ for all cell lines with respect to the control or vehicle testing conditions were observed. However, MDA-MB-231 cells display a statistically significant decrease in velocity from V_i_ to V_f_ of 40.2% resulting in a -1.21 μm/h^2^ deceleration over 24 hours. These data suggest that aligned nanofiber provides a superior substrate for cellular migration compared to randomly oriented nanofiber, yielding both the greatest total and net distance traveled. MCF-10A normal cells migrate effectively but do not respond to the applied CXCL12 gradient. With increasing metastatic potential, significant increases in total and net migration were observed in the presence of CXCL12.

**Table 2:** **Initial and final velocities of all three cell lines on random and aligned nanofibers**

Test variable	Cell type	Fiber orientation	Initial velocity (um/h) [ [t _avg_ = 1-8 h]	Final velocity (um/h) [ [t _avg_ =16-24 h]
Control	MCF-10A	Random	2.05 ± 1	1.81 ± 1
		Aligned	10.85 ± 8	10.02 ± 4
	MCF-7	Random	3.01 ± 3	4.1 ± 4
		Aligned	11.06 ± 8.4	10.86 ± 5.4
	MDA-MB-231	Random	10.85 ± 8	6.1 ± 10
		Aligned	31.6 ± 14	29.8 ± 7
Vehicle	MCF-10A	Random	2.95 ± .85	1.61 ± 1
		Aligned	11.25 ± 3	10.1 ± 3
	MCF-7	Random	2.71 ± 2	3.92 ± 2
		Aligned	10.36 ± 4	10.56 ± 2
	MDA-MB-231	Random	10.03 ± 6	8.1 ± 2
		Aligned	28.4 ± 17	25.8 ± 10
CXCL12	MCF-10A	Random	3.1 ± 5	2.0 ± 2
		Aligned	11.31 ± 4	12.1 ± 5
	MCF-7	Random	7.1 ± 5	6.0 ± 3
		Aligned	15.26 ± 13	15.82 ± 5
	MDA-MB-231	Random	11.2 ± 6.1	5.1 ± 6
		Aligned	32.3 ± 13	19.3 ± 9***

***Denotes statistical significance, p = .00001.

No significant difference was seen for any of the cell-lines early vs. late for the control or vehicle experimental conditions. MDA-MB-231 cells displayed a significant drop in velocity in presence of a CXCL12 gradient from early to late time points resulting in a -1.21 um/h^2^ deceleration, likely due to the dissipation of the gradient at later time points and inherent sensitivity of the CXCR4 receptor.

**Figure 7:**
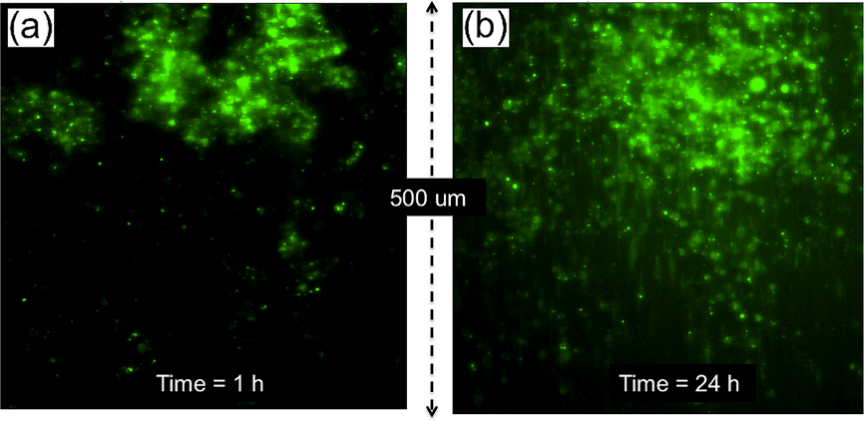
**Tumor cell migration from explanted MDA-MB-231 tumors after (a) 1 and (b) 24 hours.** (time-lapse video footage of tumor cell migration on aligned nanofiber substrates, Additional file 5).

To compare tumor cells grown *in vitro* to explanted xenograft GFP-expressing MDA-MB-231 breast tumor cells, tumors were resected from female SCID mice and applied onto aligned nanofiber to evaluate the migration potential of biopsied cells on this novel platform, *ex vivo*. Figure 7(a, b) illustrates MDA-MB-231 cells at the start of the experiment (a), and at later times (b) as they escape the tumor explant, adhering to aligned PCL nanofibers and effectively migrating in the principle direction of alignment. The spread of tumor cells is clearly evident in Figure 7(b) compared to Figure 7(a) as is the directionality of spread along the alignment, following the CXCL12 gradient (Additional file 5). Initially, tumor cells displayed rounded, spherical morphologies prior to attaching to the aligned nanofiber substrate, and a significant shift in cell morphology was observed after cell attachment on aligned nanofiber was observed with the tumor cells then displaying elongated, spindle-like morphologies. We observed a 25% increase in total distance and a 23% increase in net distance traveled for MDA-MB-231 tumor cells migrating on aligned nanofiber substrates in the presence of a CXCL12 gradient (Table 3).

**Table 3:** **Distance traveled and velocity for cells emerging from explanted MDA-MB-231 tumors with and without the CXCL-12 gradient**

	Total distance traveled (um)	Net distance traveled (um)	Velocity (um/h)
Control	200 ± 10	85 ± 7	9.25 ± 3
CXCL12	250 ± 8**	105 ± 9***	11 ± 1

**Denotes statistical significance, p = 0.001.

***Denotes statistical significant, p = 0.0001.

The gradient has no statistical effect on either velocity or net distance although the total distance traveled shows a statistically significant 25% increase. However, the velocities of cells emerging from the explanted tumors are approximately 1/3 those of seeded cells while the distances traveled are approximately ½ those of seeded cells.

### Viability and receptor expression

**Figure 8:**
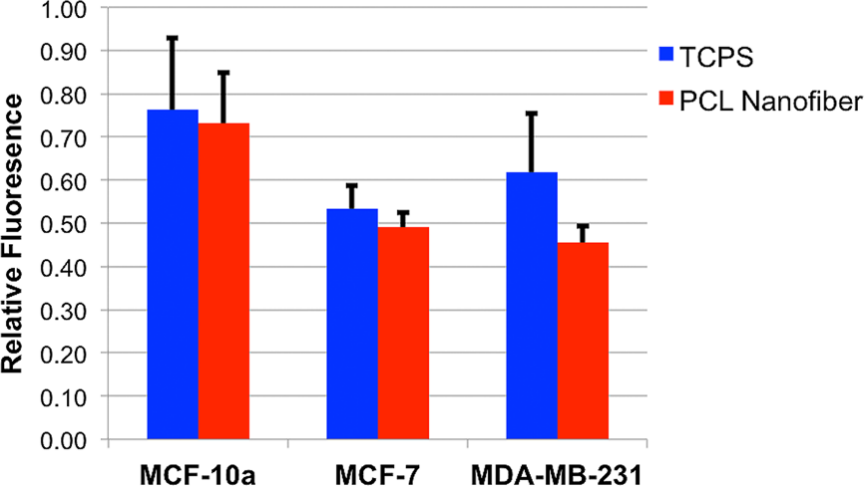
**Viability of MCF-10A, MCF-7, and MDA-MB-231 cells on TCPS vs. electrospun substrates; no statistical differences were detectable.**

Figure 8 indicates no significant difference in cell viability between MCF-10A, MCF-7, or MDA-MB-231 cells grown on fiber versus TCPS.

**Figure 9:**
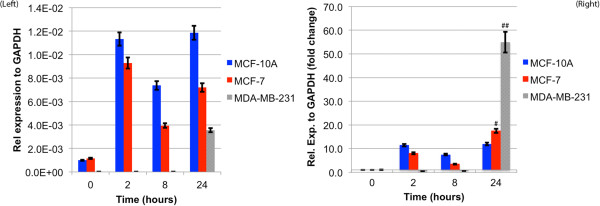
**CXCR-4 cell surface receptor expression for breast cancer cells is displayed.** (LEFT) Relative CXCR-4 expression to GAPDH versus time for the MCF-10A, MCF-7 and MDA-MB-231 cells on electrospun PCL. CXCR-4 expression for MCF-10A and MCF-7 cells prior to CXCL12 exposure display significantly greater expression compared to MDA-MB-231 cells. This trend is conserved over time post-exposure to CXCL12. (RIGHT) Fold-change in CXCR-4 expression with respect to fresh cells for MCF-10A and MCF-7 cells remains relatively constant over 24-hr time period. The MDA-MB-231 cells, however, show a dramatic reduction in CXCR-4 expression followed by a 55-fold rebound after 24 h. (#) Denotes statistically significant differences between experimental conditions, p <0.05.

To understand if differences in CXCL12-mediated cell migration were due to relative expression of CXCR4 mRNA (the receptor for CXCL12), we performed qRT-PCR for CXCR4 gene expression. MCF-10A are normal mammary epithelial cells while MCF-7 (estrogen and progesterone receptor positive, HER2 negative) and MDA-MB-231 (estrogen, progesterone, and HER2 receptor negative) cells represent two different breast cancer cell lines with increasing metastatic potential [42]. We hypothesized that sensitivity to CXCL12 gradients may differ between these cells and possibly modulate migratory potential. We cultured these cell lines in the presence of an agarose-bead containing rhCXCL12 over 24 hours. We found significant differences in basal CXCR4 mRNA expression (t = 0 hours) between the MCF-10A and MCF-7 cells (~33-fold increase) compared to MDA-MB-231 cells (p < 0.0001)) (Figure 9). Interestingly, our data indicates that CXCL12 exposure transcriptionally augments CXCR4 in both MCF-10A and MCF-7 cells as we observed an 11-fold and 7.5-fold increase, respectively, in CXCR4 mRNA expression after 2 hours. Expression patterns between these two cell lines were similar as CXCR4 mRNA expression was reduced at 8 hours (4-fold decrease for MCF10-A and 5-fold decrease for MCF-7) then again subsequently increased at 24 hours as CXCL12 was depleted (5-fold increase for MCF10-A and 15-fold decrease for MCF-7) suggesting that each MCF-10A and MCF-7 cells respond to initial exposure of CXCL12 by up-regulating CXCR4 transcription. After 8 hours exposure to CXCL12, CXCR4 transcription is suppressed. At 24 hours, when CXCL12 availability is diminished, CXCR4 transcription is once again augmented. To the contrary, the MDA-MB-231 cell CXCR4 mRNA expression profile is not augmented with initial or prolonged exposure (up to 8 hours) of CXCL12 as we observed no significant difference between time 0, 2, and 8 hours. But, at 24 hours, CXCR4 mRNA levels increase 55-fold relative to basal levels (p < 0.0002).

## Discussion

### Biomimicry and cellular morphology

Extracellular matrices are heterogeneous, 3D composites consisting of proteins arranged in fibrous morphologies. Normal breast tissue architecture is highly organized into macroscopic hollow cylindrical ducts comprised of epithelial cells organized into layered sheets anchored to a basal lamina through integrin interactions that form acinus structures [44–46]. Normal breast tissue will repair, reorganize, and remodel the epithelial lining from a polarized bilayer into a proliferative, motile, multilayered epithelium that facilitates ductal growth, maturation, and repair [9]. This normal ductal morphogenesis does not include the local dissemination of individual cells not unlike breast cancer tumorigenesis [22]. Early to late stage progression of breast cancer results in an increase in cellular proliferation within the duct, increased number of acinus, and late stage metastasis increasing desmoplasia and resulting in loss of cell-cell and basal lamina adhesion leading to stromal invasion [44, 47]. Normal mammary duct extracellular matrix (ECM) is less dense, formed mostly from collagen type I fibrils oriented randomly to support epithelium and ductal architecture [45]. The epithelial basal lamina, in contrast, is mostly composed of collagen type IV that forms thick fiber bundles oriented into aligned sheets parallel to the duct-stromal boundary [44].

**Figure 10:**
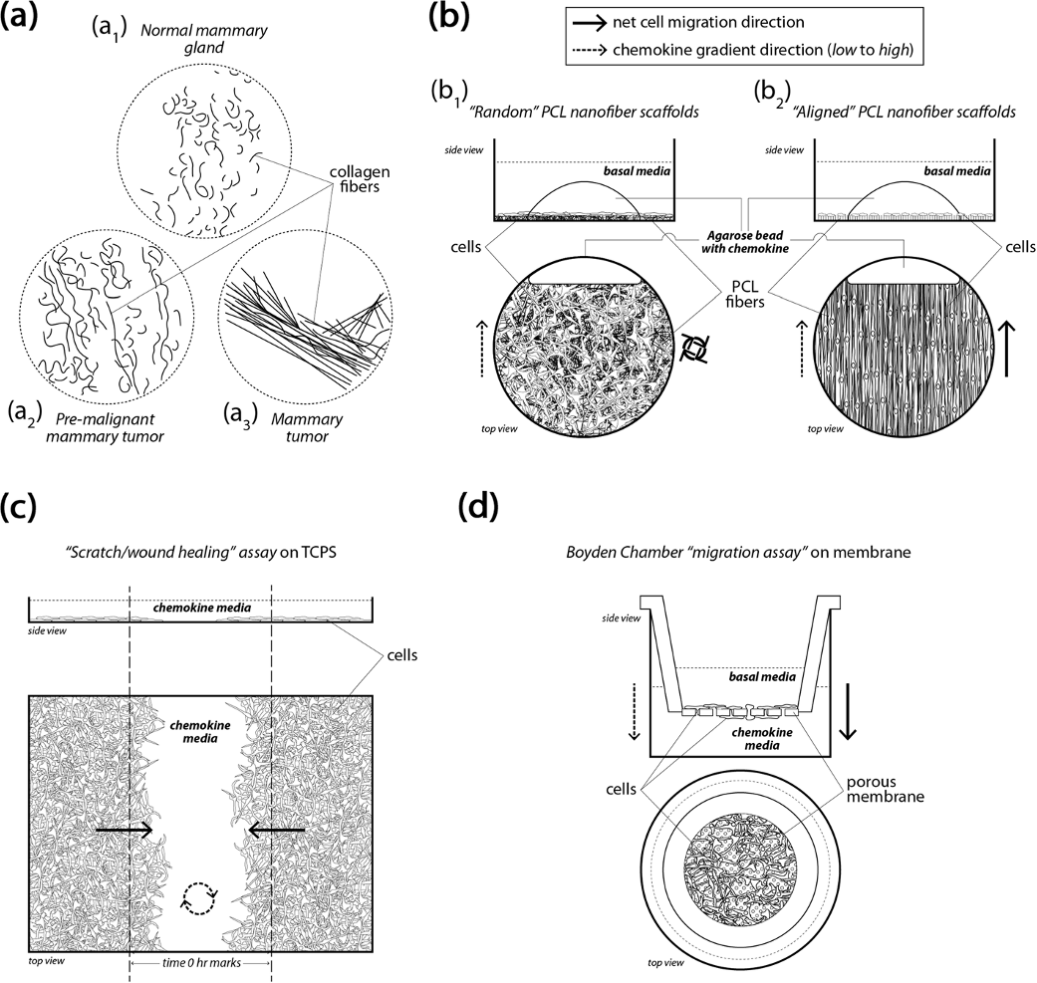
**Schemas of breast architecture at various tumor progression stages as well as common scratch or Boyden chamber in vitro assays are displayed with the developed nanofiber based migration platform.** Schematic illustrations of **(a)** normal mammary (a_1_), pre-malignant mammary tumor (a_2_), and mammary tumor collagen fiber distribution and organization (a_3_), topographically mimicked by “random” and “aligned” electrospun fiber (Figure 1(b), (c)). **(b)** Cell organization on electrospun fiber in a random organization (b_1_) resembling normal, pre-malignant mammary duct ECM; cell organization on aligned electrospun nanofibers (b_2_) recapitulates the local microenvironment at the tumor-stroma boundary of radially oriented highly aligned ECM fibrils and enhances the migratory potential of tumor cells. **(c)** The standard “scratch/wound healing” assay depicting the random movement of cells across the ‘scratch’ driven by population pressure. **(d)** The Boyden/transwell chamber migration assay in which cells migrate through 8 μm pores and appear on the other side of the membrane.

In this context we have evaluated the relative ability of electrospun nanofibers to recapitulate such structures to provide information on migration versus more traditional assays (Figure 10). The normal mammary consists of ducts displaying randomly oriented ECM fibers also observed in the random electrospun fibers (10b_1_), and as the tumor progresses it generates highly aligned ECM (as well as Figure 2) guiding attachment and migration/invasion *in vivo* also observed in the aligned electrospun fibers (10b_2_). Pre-malignant tumorgenesis is accompanied by increased collagen deposition causing crowding and disruption of the basement membranes which then gives way to morphogenesis in which the desmoplasia of transformed cells break through the basement membrane along more radially aligned ECM fibrils that aid in local invasion and migration. In contrast, both the standard scratch/wound healing assay (10c) and the Boyden Chamber (10d) consist of confluent cells adhered to 2D, flat surfaces that provide no topographical guidance. While Boyden/transwell chamber assays allow easy institution of chemotactic gradients they prevent quantitative observation of cell motility while providing quantitative assessments of cellular ability to migrate through an 8 μm pore. Finally, Table 4 succinctly summarizes the relative characteristics of each assay.

**Table 4:** **Critical comparisons of specific input and output characteristics for three different** ***in vitro*** **migration assays**

	Scratch/wound healing	Boyden/transwell	Aligned nanofiber
Sample Pool for Analysis	Confluent monolayer	Individual cells	Individual cells/tumor tissue
Biomimetic	(-)	(-)	(+)
Proliferation Dependent	(+)	(+)	(-)
Chemotaxis	(-)	(+)	(+)
Output	% of scratch remaining	Cell count	Net and total distance (μm)
Velocity Assessment	% area closed w/r/t time	NA	Velocity (μm/h)

SEM images of TCPS (Figure 1(a)) show the morphology of the substrate used for scratch assays. These substrates lack the nanofibrillar architecture of normal breast tissue (Figure 2) and cannot present topographical or orientation challenges characteristic of ECM surrounding a breast tumor [25, 31]. The random orientation of the electrospun PCL nanofibers (Figure 1(b)) better resembles the microstructure of pre-cancerous or early stage breast ECM architecture [25, 31, 45] where over-proliferation leads to increased ECM deposition causing the underlying microenvironment to become extremely dense resulting in an increase in elastic modulus [21, 48, 49]. As the ECM evolves and mutations to ductal cells arise during early-mid stage metastasis, biochemical signaling drives local stromal cells to undergo differentiation that defines them as tumor-aiding or tumor-associated cells. The most common such cells are tumor-associated macrophages (TAMS) and fibroblasts [25, 30, 50–53]. TAMs and fibroblasts allow tumor cell migration through basal lamina by increased collagen I deposition, matrix degradation, and collagen fiber reorientation [54]. TACS-3 has been shown to be a major clinical diagnostic signature of poor survival [25] and is associated with the realignment of the tumor-stromal boundary’s ECM and highly-aligned collagen fibers oriented radially or perpendicular to the tumor boundary, enhancing invasion [25, 31]. Newly formed, radially-aligned matrix acts as metastatic “cell highways” directing the migration/invasion of advanced-stage breast cancer cells providing potential for local and distant metastasis. Electrospun PCL nanofibers in Figure 1(c) closely resemble radially-aligned collagen fibril microenvironments characteristics of late-stage metastatic breast cancer [25, 31] (Figure 2). The evolution of the mammary duct’s microstructure from healthy tissue to aggressive late-stage breast cancer defines, initiates, and drives the early stages of metastasis making the physical microenvironment of a breast tumor an obligate feature of *in vitro* cell migration analysis.

Confocal images of MDA-MB-231 cells on 2D substrates (TCPS) vs. 3D substrates (electrospun nanofiber) provide insight to how cell morphology adapts to different topographies. MDA-MB-231, MCF-10A and MCF-7 cells were imaged and cellular morphology analyzed (Additional files 6, 7, 8, 9, 10, and 11). Each cell type displayed spherical shapes on TCPS accompanied by a mixture of flat or spindle-like projections. MDA-MB-231 cells displayed more spindle-like morphologies on nanofiber where the elongations followed the underlying topography. The observation of spindle-like cell-shapes as a result of significant elongation directly correlates to increased motility and migration potential [33, 55]. Underlying topography has been shown to regulate FAK and Rho/Rac genes that play an essential role in motility allowing for step-wise migration and changes in cell polarity in response to chemotactic gradients [28, 48, 56].

### Aligned fiber and phenotype

In each case, aligned PCL nanofiber allowed for greater migration distance compared to randomly-oriented nanofiber. This outcome recapitulates results in tissue biopsies from late stage breast cancer patients in which significant rearrangement of fibers oriented radially from the tumor provides direct pathways for invasion into the stroma. This observation has been termed ‘contact mediated guidance’, and is clinically associated with TACS-3 [28]. By closely observing the time-lapse video of cells on randomly-oriented nanofiber it is evident that cells still possess the ability to migrate but as the topography consists of many crossing or intersecting fibers this prevents significant progress across the matrix. Figure 1 shows MDA-MB-231 cells migrating to close the gap in a scratch assay conducted on TCPS. Time-lapse microscopy indicates that the cells close the gap in a random, wave-like fashion driven by swelling cellular populations as a result of high rates of proliferation. In contrast, *in vivo* cell metastasis is thought to be a highly-directed, orchestrated process that depends on timing, cooperation with the environment, and biochemical signaling [4, 57]. Flat, featureless TCPS has little resemblance to *in vivo* tissue ECM. In addition, Boyden/transwell assay analysis in Figure 4 depicted that the cells show very little difference in migratory capabilities without a gel present, and more pronounced effects when a gel is present. The highly porous membrane that defines the transwell assay is not a suitable barrier to assess the chemotactic migration of these cells alone. Also, the lack of quantitative outputs from this type of assay and lack of migration observation make analysis less clear. Electrospun nanofiber provides a unique platform to quantitatively and qualitatively assess cellular migration, and in these experiments include diffusion-based chemotaxis. Time-lapse microscopy tracked individual cell movements allowing for quantitative analysis of distance traveled, net distance traveled, and velocity.

In this study, cells having different levels of metastatic potential were evaluated to assess the ability of nanofiber platforms to assess migratory potential and response to CXCL12 chemotactic gradients. MCF-10A cells are normal, healthy mammary epithelial cells; while MCF-7 and MDA-MB-231 cells are breast cancer cells. MDA-MB-231 cells are late-stage, metastatic breast cancer cells known to have a high metastatic potential that we hypothesized would correlate to increased migration potential and sensitivity to chemotactic gradients. While this paper does not directly assess epithelial-to-mesenchymal transition (EMT) hallmarks, such as loss of E-cadherin, integrin signaling, and increased Rho-GTPase signaling driving cell motility and polarity for directed migration, it is thought that many of these factors might play a role in the observed results [47, 58–60]. However, as a result of passaging and growth on featureless TCPS or glass, many of the *in vivo* characteristics of these cell lines are lost. That said, an as-expected increase in migration was observed from MCF-10A to MDA-MB-231 cells, showing that with increased metastatic potential at least some of the cellular machinery associated with enhanced motility is preserved. Interestingly, this trend is not observed on random fiber, suggesting that matrix orientation and topography is as important as cellular phenotype or mutation accumulation in driving metastatic potential. The presence of CXCL12 gradients caused significant increases in total and net distance traveled for MCF-7 and MDA-MB-231 cells. However, this response was not observed on random fiber as a result of the random nanofiber topography hindering migration. An 82% increase in net distance traveled was observed for MDA-MB-231 cells, resulting in nearly 320 μm of migration. While metastasis is highly dependent on tumor vascularization and size, the presence of aligned nanofiber “highways” oriented radially from the tumor has shown clinical correlation to increased metastases and poor patient prognosis [25]. In the absence of biochemical signaling, MDA-MB-231 cells are capable of migrating 200 μm, but this motion is not as efficient as migration in the presence of a CXCL12 chemotactic gradient. MCF-10A cells only traveled more than 100 μm when CXCL12 was presented as a gradient while the control and vehicle conditions averaged only 85 μm of net migration. MCF-10A cells on average migrated 105 μm on aligned nanofiber, suggesting that these cells are capable of migrating with high efficiency but display no sensitivity to CXCL12 gradients.

### Gradient sensitivity

CXCR4 is the primary receptor for the potent chemotactic chemokine CXCL12 [61]. Many *in vivo* studies examining invasion, intravasation, and distant colonization in mouse models have shown that CXCR4 over-expressing breast cancer cells are greatly sensitive to high levels of CXCL12, creating the potential for enhanced, directed cellular migration in the direction of the gradient [62, 63]. In addition, paracrine signal loops between TAMS and tumor-aiding fibroblasts that secrete CXCL12 to CXCR4 over-expressing breast cancer cells increase the effectiveness of epidermal growth factor (EGF) gradients significantly increasing the occurrence of metastasis [64]. Conversely, compounds that block expression of CXCR4 result in a decreased anti-metastatic effect to the inhibition of leukocyte chemotaxis that relies on CXCR4/CXCL12 signaling [64, 65]. RT-PCR analysis of MCF-10A, MCF-7, and MDA-MB-231 cells showed that each of the cell lines initially express CXCR4 (Figure 9 (LEFT)), but have significantly different levels of expression and thus differences in sensitivity to the ligand CXCL12. This finding suggests that MCF-10A and MCF-7 cells are less sensitive to low levels of CXCL12 compared to MDA-MB-231 cells, likely causing a different phenotypic response to gradients of CXCL12. MCF-10A cells display less sensitivity to CXCL12 gradients resulting in no significant differences in net distance traveled compared to control or vehicle experimental conditions. MCF-7 cells display more sensitivity to CXCL12, showing that when CXCL12 is present in low concentrations the expression of CXCR4 is down regulated to modulate the response. As a result, MCF-7 cells displayed statistically significant increases in total and net distance traveled when CXCL12 was present compared to cells not exposed to the chemokine. The trend continues with increasing metastatic potential in the cell lines, showing that MDA-MB-231 cells are very sensitive to small concentrations of CXCL12 and modulate expression of CXCR4 once the gradient has dissipated in order to find the ligand. The sensitivity that MDA-MB-231 cells displayed in response to CXCL12 gradients resulted in significant increases in net distance traveled. Large standard deviations suggest that many of the cells were capable of migrating distances larger than 300 μm. However, as a result of CXCL12 sensitivity and gradient dissipation versus time, MDA-MB-231 cells displayed a deceleration with time causing significant reductions in displacement of the cells at later time points (16–24 h) as a result of the depletion of the CXCL12 gradient after 8 hours. MCF-10A cells and MCF-7 cells displayed constant or slightly positive acceleration values over the 24-hour period due to their inherently lower sensitivity to CXCL12.

The migration results from MDA-MB-231 tumor cells leaving a tumor biopsy depict trends similar to the cell lines inoculated as individual cell populations. Interestingly, significant increases in total and net distance traveled were observed. Further testing and experiments will be conducted using the nanofiber platform integrated with diffusive chemotactic gradients. Current results suggest that this platform can be an effective means of evaluating patient tumor biopsies for malignant cell populations and migration potential while speeding patient-specific evaluations of anti-metastatic compounds.

## Conclusions

While it is well understood that early detection is the best prevention of metastasis in breast cancer, tumor cells located in the margins following surgical lumpectomy or mastectomy contain populations of dormant cancer stem cells that can result in distant metastases and reduce patient survival [66–69]. Nearly 50% of patients undergoing breast tumor surgical conservation or full mastectomy therapy require a secondary excision due to positive margins [68]. Triple-negative breast cancer patients are most susceptible to recurrence and distant metastases within the first 3 years after surgical therapy even after follow-up chemotherapy or radiation [66, 67]. If novel anti-metastatic drugs are to be developed at a faster rate to prevent recurrence and relapse, *in vitro* platforms that better recapitulate the microenvironment of *in vivo* tissues must be developed. The use of electrospun nanofiber has been recently utilized for tissue engineering applications. This report has demonstrated efficacy in the use of nanofiber-based platforms to evaluate the migration and metastatic potential of breast cancer cells. The unique electrospun nanofiber microstructure and morphology mimics native ECM, and alignment can be controlled to provide either aligned or random nanofiber orientations. A combination of matrix alignment and increased migration potential of breast cancer cells allows for increased total and net migration distances not present in other assays (Table 4). Nearly 2-fold increases in net migration distance for MDA-MB-231 cells in the presence of a CXCL12 gradient suggest that drug evaluation should be conducted under conditions that optimize cell migration. This study suggests that use of a synthetic form of a nanoscale model could provide a convenient means of testing the efficacy of novel experimental anti-metastatic compounds.

## Electronic supplementary material

Additional file 1: **Plot displays the release profiles of FITC labeled BSA protein from an agarose gel over a 24-hr time period.** (PNG 16 MB)

Additional file 2: **MDA-MB-231 time-lapse video footage of cell migration on random nanofiber substrates, displaying cells reaching out filopodia attaching and anchoring to multiple fibers in multiple directions essentially preventing substantial net migration.** (MOV 179 KB)

Additional file 3: **MDA-MB-231 time-lapse video footage of cell migration on aligned nanofiber substrates, displaying highly-elongated cells with filopodia stretched out in the direction of fiber alignment allowing for and aiding migration.** (MOV 1 MB)

Additional file 4: **MDA-MB-231 time-lapse video footage of cell migration on aligned nanofiber substrates in the presence of an applied CXCL12 chemokine gradient, providing enhanced and more directed migration along fibers into an increasing chemokine concentration.** (MOV 646 KB)

Additional file 5: **Explanted GFP-labeled MDA-MB-231 breast tumor needle-biopsy from a SCID mouse displaying attachment of tumor cells (green) and ensuing migration in the direction of nanofiber alignment.** Significant dispersion of tumor cells from the biopsy in the direction of fiber alignment occurred over the 24-hr period. (MOV 792 KB)

Additional file 6: **Confocal microscopy images displaying the shape and morphology of MCF10A cells on plastic.** (TIFF 768 KB)

Additional file 7: **Confocal microscopy images displaying the shape and morphology of MCF10A cells on random nanofiber.** (TIFF 768 KB)

Additional file 8: **Confocal microscopy images displaying the shape and morphology of MCF10A cells on aligned nanofiber.** (TIFF 768 KB)

Additional file 9: **Confocal microscopy images displaying the shape and morphology of MCF7 cells on plastic.** (TIFF 768 KB)

Additional file 10: **Confocal microscopy images displaying the shape and morphology of MCF7 cells on random nanofiber.** (TIFF 768 KB)

Additional file 11: **Confocal microscopy images displaying the shape and morphology of MCF7 cells on aligned nanofiber.** (TIFF 768 KB)

## Abbreviations

SCID: Severe combined immunodeficiency
CXCL12: C-X-C motif chemokine 12
ECM: Extracellular matrix.

## References

[1] Edwards BK, Noone A, Mariotto AB, Simard EP, Boscoe FP, Henley SJ, Jemal A, Cho H, Anderson RN, Kohler BA, Eheman CR, Ward EM (2014). Annual Report to the Nation on the status of cancer, 1975-2010, featuring prevalence of comorbidity and impact on survival among persons with lung, colorectal, breast, or prostate cancer. Cancer.

[2] Abe O, Abe R, Enomoto K, Kikuchi K, Koyama H, Masuda H, Nomura Y, Sakai K, Sugimachi K, Tominaga T, Uchino J, Yoshida M, Haybittle JL, Davies C, Harvey VJ, Holdaway TM, Kay RG, Mason BH, Forbes JF, Wilcken N, Gnant M, Jakesz R, Ploner M, Yosef HMA, Focan C, Lobelle JP, Peek U, Oates GD, Powell J, Durand M (2005). Effects of chemotherapy and hormonal therapy for early breast cancer on recurrence and 15-year survival: an overview of the randomised trials. The Lancet.

[3] Boyd NF (2011). Tamoxifen, mammographic density, and breast cancer prevention. J Natl Cancer Inst.

[4] Trape AP, Gonzalez-Angulo AM (2012). Breast Cancer and Metastasis: On the Way Toward Individualized Therapy.

[5] Anders CK, Zagar TM, Carey LA (2013). The management of early-stage and metastatic triple-negative breast cancer: a review. Hematol Oncol Clin North Am.

[6] Gupta GP, Massagué J (2006). Cancer metastasis: building a framework. Cell.

[7] Chaffer CL, Weinberg RA (2011). A perspective on cancer cell metastasis. Science.

[8] Baker EL, Srivastava J, Yu D, Bonnecaze RT, Zaman MH (2011). Cancer cell migration: integrated roles of matrix mechanics and transforming potential. PLoS One.

[9] Bissell MJ, Radisky DC, Rizki A, Weaver VM, Petersen OW (2002). The organizing principle: microenvironmental influences in the normal and malignant breast. Differentiation.

[10] Dhimolea E, Soto AM, Sonnenschein C (2012). Breast epithelial tissue morphology is affected in 3D cultures by species-specific collagen-based extracellular matrix. J Biomed Mater Res Part A.

[11] Adanja I, Debeir O, Megalizzi V, Kiss R, Warzee N, Decaestecker C (2010). Automated tracking of unmarked cells migrating in three-dimensional matrices applied to anti-cancer drug screening. Exp Cell Res.

[12] Mok TSK (2011). Personalized medicine in lung cancer: what we need to know. Nat Rev Clin Oncol.

[13] Slamon DJ, Leyland-Jones B, Shak S, Fuchs H, Paton V, Bajamonde A, Fleming T, Eiermann W, Wolter J, Pegram M, Baselga J, Norton L (2001). Use of chemotherapy plus a monoclonal antibody against HER2 for metastatic breast cancer that overexpresses HER2. N Engl J Med.

[14] Tao L, Hu W, Liu Y, Huang G, Sumer BD, Gao J (2011). Shape-specific polymeric nanomedicine: emerging opportunities and challenges. Exp Biol Med.

[15] Alexander S, Friedl P (2012). Cancer invasion and resistance: interconnected processes of disease progression and therapy failure. Trends Mol Med.

[16] Mahamodhossen YA, Liu W, Rong-Rong Z (2013). Triple-negative breast cancer: new perspectives for novel therapies. Med Oncol.

[17] Noonan DM, Pennesi G, Albini A (2010). Invasion and Metastasis.

[18] Shibue T, Weinberg RA (2011). Metastatic colonization: settlement, adaptation and propagation of tumor cells in a foreign tissue environment. Semin Cancer Biol.

[19] Spano D, Zollo M (2012). Tumor microenvironment: a main actor in the metastasis process. Clin Exp Metastasis.

[20] Friedl P, Alexander S (2011). Cancer invasion and the microenvironment: plasticity and reciprocity. Cell.

[21] Hansen RK, Bissell MJ (2000). Tissue architecture and breast cancer: the role of extracellular matrix and steroid hormones. Endocr Relat Cancer.

[22] Nguyen-Ngoc K-V, Cheunga KJ, Brenot A, Shamir ER, Gray RS, Hines WC, Yaswen P, Werb Z, Ewald AJ (2012). ECM microenvironment regulates collective migration and local dissemination in normal and malignant mammary epithelium. Proc Natl Acad Sci U S A.

[23] Alexander S, Weigelin B, Winkler F, Friedl P (2013). Preclinical intravital microscopy of the tumour-stroma interface: invasion, metastasis, and therapy response. Curr Opin Cell Biol.

[24] Gritsenko PG, Ilina O, Friedl P (2012). Interstitial guidance of cancer invasion. J Pathol.

[25] Conklin MW, Eickhoff JC, Riching KM, Pehlke CA, Eliceiri KW, Provenzano PP, Friedl A, Keely PJ (2011). Aligned collagen is a prognostic signature for survival in human breast carcinoma. Am J Pathol.

[26] Ilina O, Bakker G, Vasaturo A, Hofmann RM, Friedl P (2011). Two-photon laser-generated microtracks in 3D collagen lattices: principles of MMP-dependent and -independent collective cancer cell invasion. Phys Biol.

[27] Provenzano PP, Eliceiri KW, Inman DR, Keely PJ (2010). Engineering Three-Dimensional Collagen Matrices to Provide Contact Guidance During 3D Cell Migration.

[28] Provenzano PP, Inman DR, Eliceiri KW, Trier SM, Keely PJ (2008). Contact guidance mediated three-dimensional cell migration is regulated by Rho/ROCK-dependent matrix reorganization. Biophys J.

[29] Tamimi SO, Ahmed A (1987). Stromal changes in invasive breast-carcinoma - an ultrastructural-study. J Pathol.

[30] Conklin MW, Keely PJ (2012). Why the stroma matters in breast cancer insights into breast cancer patient outcomes through the examination of stromal biomarkers. Celll Adhes Migr.

[31] Provenzano PP, Eliceiri KW, Campbell JM, Inman DR, White JG, Keely PJ (2006). Collagen reorganization at the tumor-stromal interface facilitates local invasion.

[32] Abraham LC, Dice JF, Finn PF, Mesires NT, Lee K, Kaplan DL (2007). Extracellular matrix remodeling--methods to quantify cell-matrix interactions. Biomaterials.

[33] Agudelo-Garcia PA, De Jesus JK, Williams SP, Nowicki MO, Chiocca EA, Liyanarachchi S, Li PK, Lannutti JJ, Johnson JK, Lawler SE, Viapiano MS (2011). Glioma cell migration on three-dimensional nanofiber scaffolds is regulated by substrate topography and abolished by inhibition of STAT3 signaling. Neoplasia.

[34] Eisenberg MC, Kim Y, Li R, Ackerman WE, Kniss DA, Friedman A (2011). Mechanistic modeling of the effects of myoferlin on tumor cell invasion. PNAS.

[35] Haessler U, Teo JCM, Foretay D, Renaud P, Swartz MA (2012). Migration dynamics of breast cancer cells in a tunable 3D interstitial flow chamber. Integr Biol.

[36] Johnson J, Nowicki MO, Lee CH, Chiocca EA, Viapiano MS, Lawler SE, Lannutti JJ (2009). Quantitative analysis of complex glioma cell migration on electrospun polycaprolactone using time-lapse microscopy. Tissue Eng Part C: Methods.

[37] Rao SS, Nelson MT, Xue R, DeJesus JK, Viapiano MS, Lannutti JJ, Sarkar A, Winter JO (2013). Mimicking white matter tract topography using core-shell electrospun nanofibers to examine migration of malignant brain tumors. Biomaterials.

[38] Beachley V, Wen XJ (2009). Effect of electrospinning parameters on the nanofiber diameter and length. Mater Sci Eng C.

[39] Johnson J, Ghosh A, Lannutti J (2007). Microstructure-property relationships in a tissue-engineering scaffold. J Appl Polym Sci.

[40] Powell HM, Boyce ST (2009). Engineered Human Skin Fabricated Using Electrospun Collagen-PCL Blends: Morphogenesis and Mechanical Properties.

[41] Schnell E, Klinkhammer K, Balzer S, Brook G, Klee D, Dalton P, Mey J (2007). Guidance of glial cell. migration and axonal growth on electrospun nanofibers of poly-epsilon-caprolactone and a collagen/poly-epsilon-caprolactone blend. Biomaterials.

[42] Holliday DL, Speirs V (2011). Choosing the right cell line for breast cancer research. Breast Cancer Res.

[43] Gaumer J, Prasad A, Lee D, Lannutti J (2009). Source-to-ground distance and the mechanical properties of electrospun fiber. Acta Biomater.

[44] Nam J, Huang Y, Agarwal S, Lannutti J (2008). Materials selection and residual solvent retention in biodegradable electrospun fibers. J Appl Polym Sci.

[45] Bissell MJ, Rizki A, Mian IS (2003). Tissue architecture: the ultimate regulator of breast epithelial function - commentary. Curr Opin Cell Biol.

[46] Andrews JL, Kim AC, Hens JR (2012). The role and function of cadherins in the mammary gland. Breast Cancer Res.

[47] Raviraj V, Zhang H, Chien H, Cole L, Thompson EW, Soon L (2012). Dormant but migratory tumour cells in desmoplastic stroma of invasive ductal carcinomas. Clin Exp Metastasis.

[48] Provenzano PP, Inman DR, Eliceiri KW, Keely PJ (2009). Matrix density-induced mechanoregulation of breast cell phenotype, signaling and gene expression through a FAK-ERK linkage. Oncogene.

[49] Provenzano PP, Inman DR, Eliceiri KW, Knittel JG, Yan L, Rueden CT, White JG, Keely PJ (2008). Collagen density promotes mammary tumor initiation and progression. BMC Med.

[50] Eubank TD, Roberts RD, Khan M, Curry JM, Nuovo GJ, Kuppusamyl P, Marsh CB (2009). Granulocyte macrophage colony-stimulating factor inhibits breast cancer growth and metastasis by invoking an anti-angiogenic program in tumor-educated macrophages. Cancer Res.

[51] Guiet R, Van Goethem E, Cougoule C, Balor S, Valette A, Al Saati T, Lowell CA, Le Cabec V, Maridonneau-Parini I (2011). The Process of Macrophage Migration Promotes Matrix Metalloproteinase-Independent Invasion by Tumor Cells. J Immunol.

[52] Ingman WV, Wyckoff J, Gouon-Evans V, Condeelis J, Pollard JW (2006). Macrophages Promote Collagen Fibrillogenesis Around Terminal End Buds of the Developing Mammary Gland.

[53] Krol M, Pawlowski KM, Majchrzak K, Dolka I, Abramowicz A, Szyszko K, Motyl T (2011). Density of tumor-associated macrophages (TAMs) and expression of their growth factor receptor MCSF-R and CD14 in canine mammary adenocarcinomas of various grade of malignancy and metastasis. Pol J Vet Sci.

[54] Chung AS, Waldeck H, Schmidt DR, Kao WJ (2009). Monocyte inflammatory and matrix remodeling response modulated by grafted ECM-derived ligand concentration. J Biomed Mater Res A.

[55] Koch TM, Muenster S, Bonakdar N, Butler JP, Fabry B (2012). 3D traction forces in cancer cell invasion. PLoS One.

[56] Kumar G, Co CC, Ho C (2011). Steering cell migration using microarray amplification of natural directional persistence. Langmuir.

[57] Horimoto Y, Polanska UM, Takahashi Y, Orimo A (2012). Emerging roles of the tumor-associated stroma in promoting tumor metastasis. Cell Adh Migr.

[58] Scheel C, Weinberg RA (2012). Cancer stem cells and epithelial-mesenchymal transition: concepts and molecular links. Semin Cancer Biol.

[59] Takebe N, Warren RQ, Ivy SP (2011). Breast cancer growth and metastasis: interplay between cancer stem cells, embryonic signaling pathways and epithelial-to-mesenchymal transition. Breast Cancer Res.

[60] Yao D, Dai C, Peng S (2011). Mechanism of the mesenchymal-epithelial transition and its relationship with metastatic tumor formation. Mol Cancer Res.

[61] Jin F, Brockmeier U, Otterbach F, Metzen E (2012). New insight into the SDF-1/CXCR4 axis in a breast carcinoma model: hypoxia-induced endothelial SDF-1 and tumor cell CXCR4 are required for tumor cell intravasation. Mol Cancer Res.

[62] Boimel PJ, Smirnova T, Zhou ZN, Wyckoff J, Park H, Coniglio SJ, Qian B, Stanley ER, Cox D, Pollard JW, Muller WJ, Condeelis J, Segall JE (2012). Contribution of CXCL12 secretion to invasion of breast cancer cells. Breast Cancer Res.

[63] Williams SA, Harata-Lee Y, Comerford I, Anderson RL, Smyth MJ, McColl SR (2010). Multiple functions of CXCL12 in a syngeneic model of breast cancer. Mol Cancer.

[64] Zhuo W, Jia L, Song N, Lu X, Ding Y, Wang X, Song X, Fu Y, Luo Y (2012). The CXCL12-CXCR4 Chemokine Pathway: A Novel Axis Regulates Lymphangiogenesis. Clin Cancer Res.

[65] De Oliveira KB, Guembarovski RL, Fiorina Losi Guembarovski AM, da Silva do Amaral Herrera AC, Jorge Sobrinho W, Ariza CB, Ehara Watanabe MA (2013). CXCL12, CXCR4 and IFN gamma genes expression: implications for proinflammatory microenvironment of breast cancer. Clin Exp Med.

[66] Arriagada R, Lê MG, Contesso G, Guinebretière JM, Rochard F, Spielmann M (2002). Predictive factors for local recurrence in 2006 patients with surgically resected small breast cancer. Ann Oncol.

[67] Dent R, Trudeau M, Pritchard KI, Hanna WM, Kahn HK, Sawka CA, Lickley LA, Rawlinson E, Sun P, Narod SA (2007). Triple-negative breast cancer: clinical features and patterns of recurrence. Clin Cancer Res.

[68] Haka AS, Volynskaya Z, Gardecki JA, Nazemi J, Lyons J, Hicks D, Fitzmaurice M, Dasari RR, Crowe JP, Feld MS (2006). In vivo margin assessment during partial mastectomy breast surgery using raman spectroscopy. Cancer Res.

[69] Muehlberg FL, Song Y, Krohn A, Pinilla SP, Droll LH, Leng X, Seidensticker M, Ricke J, Altman AM, Devarajan E, Liu W, Arlinghaus RB, Alt EU (2009). Tissue-resident stem cells promote breast cancer growth and metastasis. Carcinogenesis.

[CR70] The pre-publication history for this paper can be accessed here:http://www.biomedcentral.com/1471-2407/14/825/prepub

