# Supplementary figures and images for: Preferential, enhanced breast cancer cell migration on biomimetic electrospun nanofiber ‘cell highways’
Source: BMC Cancer. 2014 Nov 10;14:825. doi: 10.1186/1471-2407-14-825 (PMC4236463; doi:10.1186/1471-2407-14-825)

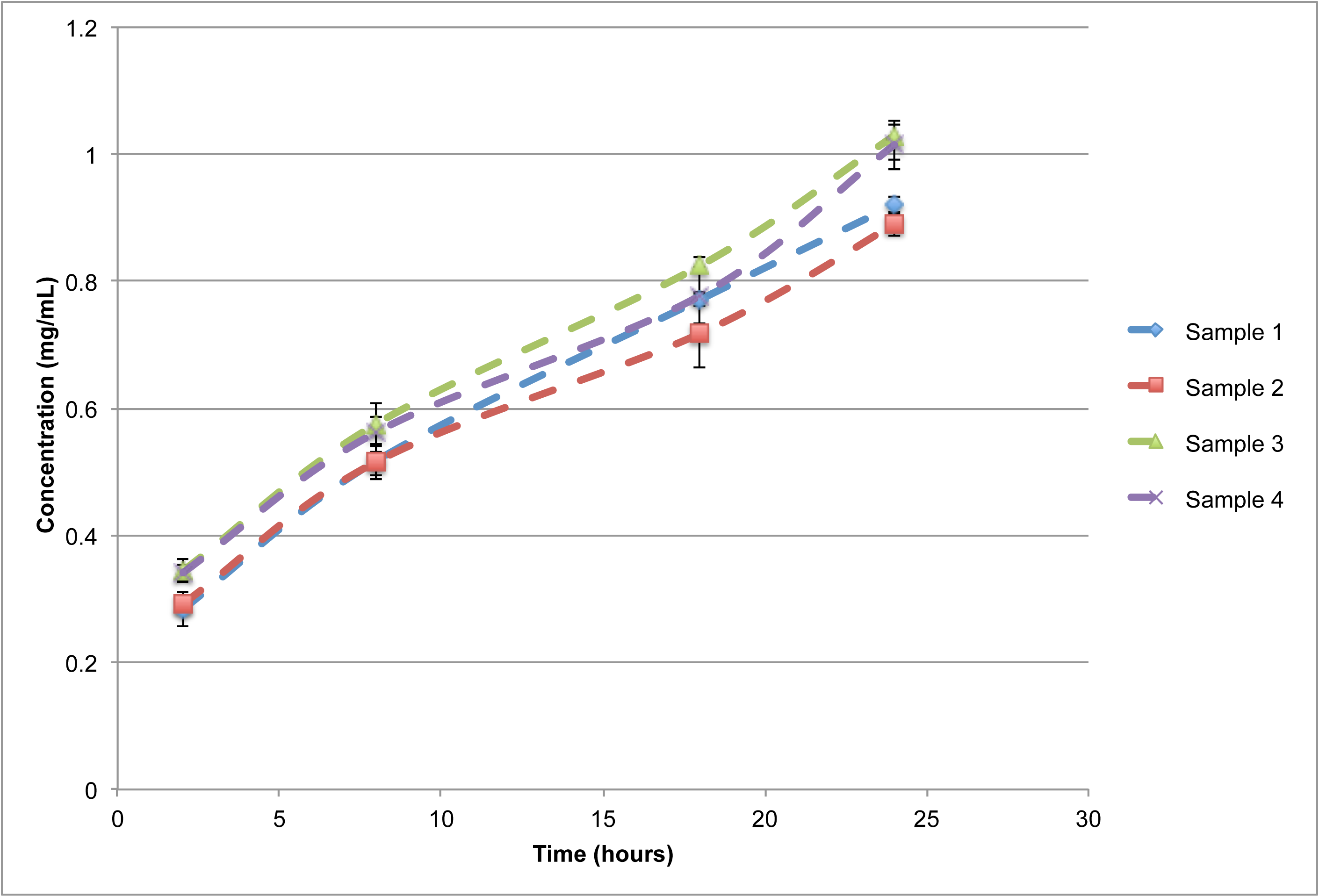

Supplement: Supplementary file 1 — Additional file 1: Plot displays the release profiles of FITC labeled BSA protein from an agarose gel over a 24-hr time period. (PNG 16 MB) [file 12885_2014_5003_MOESM1_ESM.png]

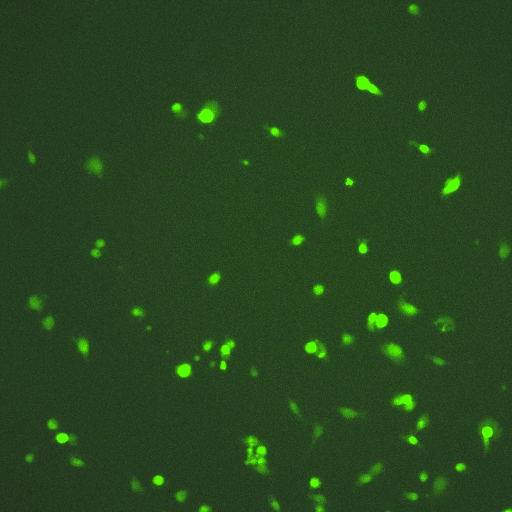

Supplement: Supplementary file 6 — Additional file 6: Confocal microscopy images displaying the shape and morphology of MCF10A cells on plastic. (TIFF 768 KB) [file 12885_2014_5003_MOESM6_ESM.tiff]

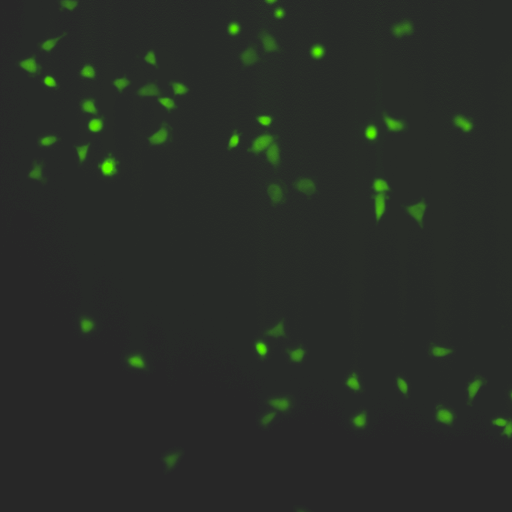

Supplement: Supplementary file 7 — Additional file 7: Confocal microscopy images displaying the shape and morphology of MCF10A cells on random nanofiber. (TIFF 768 KB) [file 12885_2014_5003_MOESM7_ESM.tiff]

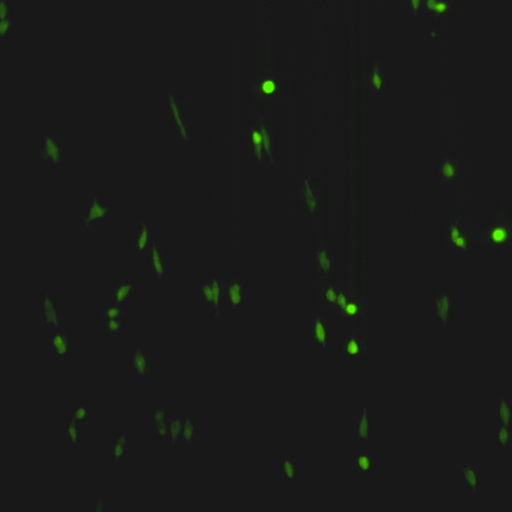

Supplement: Supplementary file 8 — Additional file 8: Confocal microscopy images displaying the shape and morphology of MCF10A cells on aligned nanofiber. (TIFF 768 KB) [file 12885_2014_5003_MOESM8_ESM.tiff]

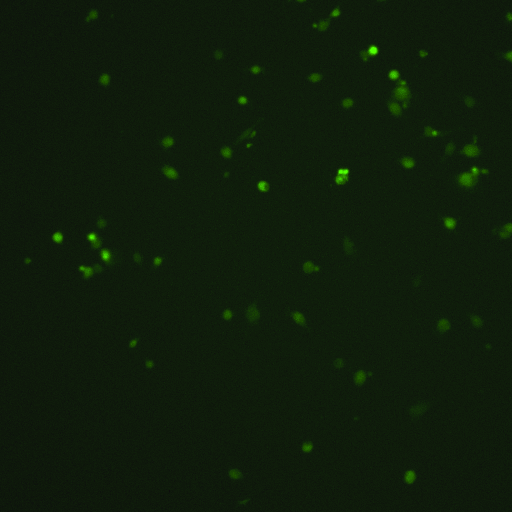

Supplement: Supplementary file 9 — Additional file 9: Confocal microscopy images displaying the shape and morphology of MCF7 cells on plastic. (TIFF 768 KB) [file 12885_2014_5003_MOESM9_ESM.tiff]

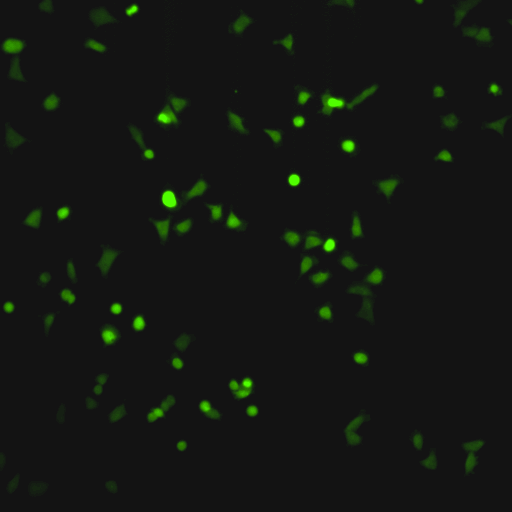

Supplement: Supplementary file 10 — Additional file 10: Confocal microscopy images displaying the shape and morphology of MCF7 cells on random nanofiber. (TIFF 768 KB) [file 12885_2014_5003_MOESM10_ESM.tiff]

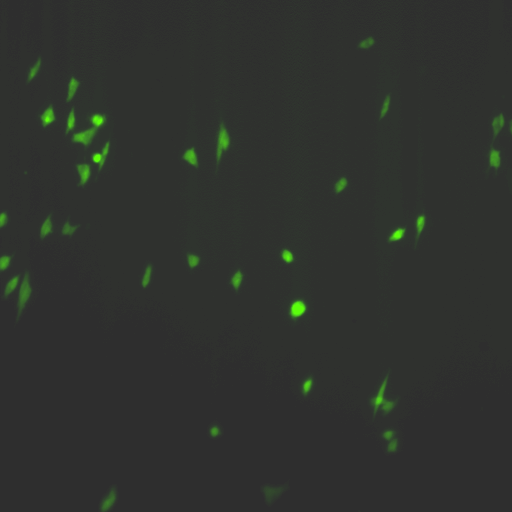

Supplement: Supplementary file 11 — Additional file 11: Confocal microscopy images displaying the shape and morphology of MCF7 cells on aligned nanofiber. (TIFF 768 KB) [file 12885_2014_5003_MOESM11_ESM.tiff]
